# Modulating the gut microbiota in Crohn’s disease: a pilot study on the impact of a plant-based diet with DNA-based monitoring

**DOI:** 10.3389/fnut.2024.1502967

**Published:** 2024-10-31

**Authors:** Stine Karstenskov Østergaard, Zeynep Cetin, Henrik Højgaard Rasmussen, Helle Nygaard Lærke, Mette Holst, Charlotte Lauridsen, Jeppe Lund Nielsen

**Affiliations:** ^1^Department of Chemistry and Bioscience, Aalborg University, Aalborg, Denmark; ^2^Department of Gastroenterology and Hepatology, Center for Nutrition and Intestinal Failure, Aalborg University Hospital, Aalborg, Denmark; ^3^Department of Clinical Medicine, Aalborg University, Aalborg, Denmark; ^4^The Dietitians and Nutritional Research Unit, EATEN, Copenhagen University Hospital, Copenhagen, Denmark; ^5^Department of Animal and Veterinary Sciences, Aarhus University, Foulum, Denmark

**Keywords:** TRNL, 16S rRNA, DNA-based dietary monitoring, plant-based diet, Crohns disease, diet intervention

## Abstract

**Introduction:**

Crohn’s Disease (CD) is characterized by chronic intestinal inflammation and dysbiosis. This study aimed to investigate the effects of a plant-based diet (PBD) on gut microbiota composition and inflammation in CD patients and assess the utility of *trn*L gene sequencing for monitoring dietary adherence.

**Methods:**

Fourteen CD patients participated in a 12-week PBD intervention. Dietary adherence was monitored through self-reported food diaries and trnL sequencing, which detects plant residues in fecal samples. Gut microbiota was analyzed using 16S rRNA sequencing, and fecal calprotectin levels were measured as an indicator of intestinal inflammation.

**Results:**

*Trn*L sequencing identified 55 plant genera in fecal samples, compared to 41 reported in food diaries, highlighting its accuracy in assessing plant residue diversity. By week 4, participants demonstrated a 1.4-fold increase in plant intake, correlating with a significant increase in microbial diversity. Key genera associated with gut health, such as *Faecalibacterium* and *Bacteroides*, increased in abundance. Additionally, fecal calprotectin levels decreased from 472 mg/kg at baseline to 207 mg/kg at week 12, indicating reduced intestinal inflammation.

**Discussion:**

A PBD positively influenced gut microbiota composition and decreased intestinal inflammation in CD patients. The study also demonstrated that *trn*L sequencing is an effective tool for assessing dietary adherence in clinical settings, offering a more objective measure than self-reported food diaries.

## 1 Introduction

Crohn’s disease (CD) is a chronic relapsing disorder mainly affecting the gastrointestinal (GI) tract and is characterized by discontinuous transmural inflammation that may affect the entire GI tract (1). The etiology of CD remains to be elucidated but the complex interplay between genetic predisposition, immune dysregulation, environmental factors, and the gut microbiome is believed to contribute to its intricate pathogenesis (2). Dysbiosis, encompassing loss of beneficial microbes, diversity, and expansion of pathobionts (3), is increasingly considered to be causative in relation to the CD pathogenesis (4). While medical interventions, including immunomodulatory drugs and biologics, have significantly improved symptom management (5), it has been suggested that due to the well-recognized modulating effect of diet on the enteric microbiome, diet-interventions hold potential as an anti-inflammatory strategy to mitigate the dysbiotic microbiome observed in CD (6, 7). Most current treatments are directed against the dysregulated immune response failing to counteract possible environmental fluctuations such as changes in the dysbiotic microbiome. With remission rates below 50% (2), research into diet as a complementary treatment to induce and maintain long-term remission holds great potential.

The human diet serves as a crucial determinant in shaping the composition and functionality of the gut microbiota. Dietary components act as substrates for microbial metabolism, influencing the abundance of specific bacterial taxa and the production of various microbial metabolites (8). Short-chain fatty acids (SCFAs), such as acetate, propionate, and butyrate, are produced by intestinal bacteria from dietary fibers and other undigested substrates entering the large intestine. This production is one of the benefits humans gain from their symbiotic relationship with gut bacteria (4). SCFAs have a large repertoire of functions ranging from regulation of ion absorption and gut motility to regulation of epithelial cells to maintain intestinal homeostasis (4, 9). A decrease in SCFA-producing bacteria such as *Dialister Invisus* and *Faecalibacterium Prausnitzii* (10) as well as fecal SCFA levels have been associated with CD (11). However, SCFAs are not a unique example. Dietary components are constantly modulated and metabolized by enteric bacteria. Diets rich in fibers, polyphenols, and prebiotics can promote the growth of anti-inflammatory microbial communities, while diets high in fat and protein (such as meat) but low in fiber can lead to dysbiosis, creating an environment that favors the expansion of pro-inflammatory microbes (6). The latter, often referred to as a Western or Westernized diet, has in recent decades spread from high-to low-income countries (12) and in conjunction so has the incidence of inflammatory diseases in these regions (13). In fact, a continuous consumption of a Westernized diet alters enteric microbiota and disrupts the epithelial barrier initiating low-grade inflammation (14). Intriguingly, a high dietary intake of fiber reduces the risk of CD by 40% (15). This highlights the bidirectional relationship where dietary choices influence the microbial composition, and in turn, the microbiota impacts the disease course.

Although diet is the most ubiquitous environmental factor known to contribute to the structure of the enteric microbiome (16), it remains poorly understood how microbes and the small molecules they modulate may interact to cause, sustain, or mitigate CD. Several review articles have summarized the research on diet concerning CD (17–19). While studies have investigated the effect of single nutrients, these are often with limited or inconclusive results. This might be attributed to the lack of understanding of how nutrients interact in the entire food matrix (8) demonstrating the need for research into entire dietary patterns to elucidate possible effects on the microbiome and CD. With pediatric CD patients, recent trials have demonstrated dietary interventions as an effective treatment. The tested diets included a Specific Carbohydrate Diet (SCD), a modified SCD (mSCD, including oats), the Crohn’s disease exclusion diet (CDED) with partial enteral nutrition (PEN), and an exclusive enteral nutrition (EEN) diet (20–26). Both SCD and mSCD induced remission in 100% of the children in a randomized trial (26), while the CDED+PEN or EEN resulted in remission rates >63% (25). The dietary intervention led to microbial changes, including an increase in the abundance of *Faecalibacterium prausnitzii, Roseburia hominis*, and *Eubacterium eligens* (23, 25, 26). Although dietary intervention trials with adult CD patients do not show remission rates similar to those of children, the SCD and a mediterranean diet induced a remission rate of >50% with a reduction of fecal calprotectin levels of ≥30%. The Inflammatory Bowel Disease-Anti-Inflammatory Diet (IBD-AID) is another diet tested at pilot scale and is developed to restore eubiosis in patients with IBD by increasing the intake of pre-and probiotics foods and reducing food known to trigger inflammation. This diet was correlated with an increase in the abundance of *Clostridia* and *Bacteriodes* often depleted in IBD patients (27). Interestingly, a plant-based diet (PBD) has recently been recommended for IBD (28), followed up by a study demonstrating that the induction rate of remission was highest in patients receiving a combination of biologic treatment and PBD (29). With known and suggested positive effects of PBD, the feasibility of introducing a PDB to CD patients in biological treatment was recently investigated (30) and the study demonstrated that it was possible to retain almost all patients during the 12-week intervention without major adverse effects.

In this study, our primary outcome was to characterize the fecal microbiota in relation to plant intake and fecal calprotectin during a 12-week PBD intervention of CD patients in remission or with mild to moderate activity to investigate possible correlations with fecal calprotectin as a biomarker of inflammation. The rationale for choosing a 12-week intervention period lies in the potential for observable changes within a defined timeframe. This duration allows for an exploration of both short-term responses and the ability to maintain any positive effects of introducing a PBD over a longer period. Most previous studies investigating the role of different diets and food components on the enteric microbiome rely on food questionnaires in combination with a sequencing technique. These dietary records, although informative, might lack important information about the actual dietary composition (31), which can be elucidated by, e.g., DNA-based surveillance methods (e.g., markers specifically targeting plants). Furthermore, the inclusion of CD patients currently undergoing biological treatments with mild to moderate symptoms enhances the clinical relevance of the study, addressing a subgroup that may particularly benefit from adjunct dietary strategies.

## 2 Methods

### 2.1 Ethical statement and patient enrollment

This study, approved by The North Denmark Region Committee on Health Research Ethics (N-20220049), involved a cohort of 15 patients over 18 years old, diagnosed with CD for more than 6 months. The patients were recruited at Aalborg University Hospital between January 8 and April 2, 2023. The study population has previously been described in detail (30). Briefly, the 15 patients were recruited to investigate the feasibility of introducing a plant-based diet to patients receiving biological treatment during remission and/or with moderate disease activity as determined by the Crohn’s Disease Activity Index (CDAI <150) (32). Diversity among the participants was sought regarding age, gender, years of receiving biological treatment, and educational background. Participants received free pre-tailored evening meals from a meal delivery provider for the entire household during the 12-week intervention as well as guidance in the transition in dietary lifestyle especially in relation to sufficient protein intake. Seafood was optional twice a week. The patients were monitored closely during the intervention. At baseline (week 0), week 4, and week 12 of the intervention, blood samples (after 4 h fasting) and fecal samples were collected and stored at −80°C until further processing. Three days prior to each sample collection, the participants filled out food diaries. The study was conducted as a prospective, single-arm study where the participants’ baseline samples were used as their own control. Participation in the intervention was voluntary and all recruited patients signed a written informed consent prior to commencement.

### 2.2 Fecal calprotectin

Fecal calprotectin levels were measured at Aalborg University Hospital, Department of Clinical Biochemistry using the Fcal turbo kit (Bühlmann, Switzerland) and analyzed using immunoturbidimetry on an Atellica (Siemens, Germany).

### 2.3 DNA extraction, amplicon generation, and sequencing

Total genomic DNA was extracted using the DNeasy® PowerLyzer PowerSoil kit (Qiagen, Germany) following the manufacturer’s specifications and eluted in 100 μL C6 solution. For bacterial detection, the 16S rRNA gene was targeted using primers specific to the V1-V8 region, while plant residues were identified through amplification of the chloroplast *trn*L gene. Additionally, the mitochondrial cytochrome b gene was used to detect porcine and bovine DNA in the samples (Table 1). The PCR-reactions was conducted in duplicates of 25 μL (PCRBIO 1x Ultra Mix (PCR BIOSYSTEMS, United Kingdom), 400 nM of each primer, 10 ng of template DNA, and nuclease-free water) and included an initial denaturation at 95°C for 2 min and a final elongation at 72°C for 5 min.

**Table 1:** Primers used for amplicon generation.

Primers	Primer sequence (5′-3′)	Amplicon length (bp)	PCR conditions	Cycles	Sample:bead ratio	Reference(s)
16S rRNA gene (Bacteria) 27F 1392R	AGRGTTYGATYMTGGCTCAG GACGGGCGGTGWGTRCA	1,365	15 s at 95°C 15 s at 55°C 90 s at 72°C	30	0.7	(53, 54)
Chloroplast intron of the *trn*L (UAA) gene (Plants) c d	CGAAATCGGTAGACGCTACG GGGGATAGAGGGACTTGAAC	200–700	15 s at 95°C 15 s at 50°C 50 s at 72°C	35	0.9	(41)
Mitochondrial cytochrome b gene (Porcine and Bovine) SIM_uni_F Pig_R^1^ Cattle_R^2^	GACCTCCCAGCTCCATCAAACATCTC-ATCTTGATGAAA GCTGATAGTAGATTTGTGATGACCGTA CTAGAAAAGTGTAAGACCCGTAATATA-AG	398^1^ 274^2^	1: 15 s at 95°C 15 s at 60°C 30 s at 72°C 2: 15 s at 95°C 15 s at 56°C 30 s at 72°C	35 35	0.8 0.8	(55)

The quality of amplicon generation was ensured by including a positive and negative control in the PCR reaction. The libraries were purified using CleanNGS (CleanNA, the Netherlands) using a sample:bead ratio specific for the amplicon (Table 1) and eluted in 25 μL of nuclease-free water. The size of selected libraries was checked by Agilent 4,150 TapeStation using ScreenTape D1000/D5000 (Agilent Technologies, United States).

The PCR products were barcoded and subsequently pooled equimolarly. The pooled libraries were DNA repaired and end-prepped, adapter ligated, cleaned and 50 fmol loaded onto a MinION R10.4.1 flow cell using the SQK-LSK114 with the EXP-PBC096 in accordance with manufacturer’s recommendations (Oxford Nanopore Technologies, United Kingdom). The library was sequenced for 72 h.

All DNA concentrations were measured using Qubit™ 1x dsDNA HS Assay Kit (Invitrogen, United States) on Qubit 4 fluorometer (Invitrogen, United States).

### 2.4 Pre-processing of amplicon sequencing data

Raw reads were basecalled and demultiplexed using Dorado v.0.5.0 with sup v.4.3.0^1^ with standard settings in the MinKNOW software with the addition of require barcodes in both ends to ensure accurate assignment. Amplicon pre-processing was conducted using the ONT-AmpSeq pipeline (33) with Q-score = 20 and filtered according to respective lengths (Table 1). The OTU’s originating from amplification of the 16S rRNA gene were taxonomically classified in VSEARCH using Midas as a reference database (34). The OTU’s originating from the mitochondrial cytochrome b and *trn*L gene were mapped using the blastn algorithm, BLAST+ v2.15.0 (35). The complete amplicon pipeline can be found at https://github.com/MathiasEskildsen/ONT-AmpSeq.

### 2.5 Mathematical modeling and statistical analysis

The resulting OTUs were analyzed in R v. 4.4.0 (36) through Rstudio v. 2024.04.2 (37). Rarefaction curves, alpha diversity plots and ordination plots were made using the ampvis2 package v. 2.8.9 (38). The Chao1 diversity index visualized using the ggplot2 package (39). The Wilcoxon ranked sum test and Kruskal-Wallis was used to investigate statistically significant (*p* < 0.05) differences. MaAsLin2 (40) was used to determine linear associations between the microbial community and para-clinical markers across the three collection points.

## 3 Results

### 3.1 Demographics of study participants and retention rate

Fifteen patients were enrolled at baseline as previously described (30). Briefly, two patients dropped out before the follow up at week 4 and were therefore excluded. Two additional patients entered the study 3 weeks after baseline keeping the cohort at 15 patients. One patient did not reach the 12-week follow up due to hospitalization, leaving a total of 14 patients completing the intervention. All participants received biological treatment with 73.3% receiving Infliximab (Table 2).

**Table 2:** Demographics of all included patients at baseline.

CD patients included in analysis (*n* = 15)	*n* (%) or mean ± SD
Average age (years)	50.7 ± 15.4
In biological treatment	15 (100%)
Infliximab	11 (73.3%)
Diagnosis before age 40	8 (53.3%)
Smoking	2 (13.3%)
Alcohol consumption per week (14 grams per unit)	4.2 ± 3 units
Family history of IBD	5 (33.3%)
Disease location	
Ileal	7 (46.7%)
Colonic	5 (33.3%)
Ileocolonic	3 (20%)

### 3.2 Diversity in plant intake significantly increased during the PBD intervention

**Figure 1:**
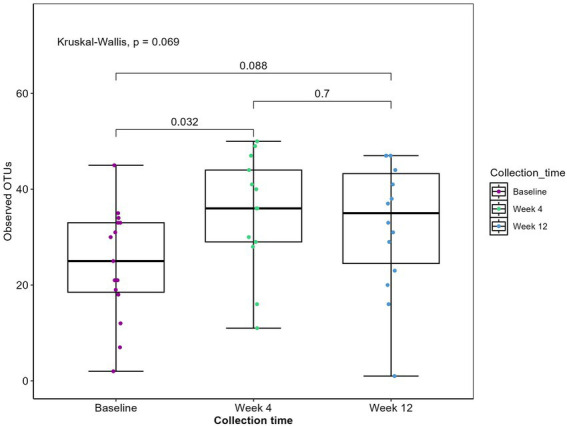
Observed plant genera richness. Boxplot displaying observed plant genera richness. Each dot represents a sample. The boxplots show the median, the first quantile and the third quantile and the whiskers extent to 1.5 interquartile range (IQR). *p*-values are determined using the Wilcoxon rank sum test and Kruskal-Wallis.

A total of 44 fecal samples were collected during the intervention: 15 at baseline, 15 at week 4, and 14 at week 12. Sequencing of the *trn*L gene resulted in a total of 3,021,168 reads with an average sequencing depth of 68,662 ± 29,099 (mean ± SD). To evaluate the plant residue diversity, all detected plants were clustered at the genus level to avoid misclassification (41). At baseline, the median intake of plant genera among all patients was 24 (Figure 1). By week 4, the median intake of plant genera had increased 1.4-fold (*p* = 0.032) to 35, indicating adherence to the diet and an expanded repertoire of plant consumption. Changes in plant richness were observed in the first 4 weeks of the intervention with no additional increase in the remaining intervention. As two patients entered the study 3 weeks into the intervention, it was tested whether their removal from the dataset would affect alpha diversity. The results indicated no significant difference in plant genera alpha diversity with their exclusion (data not shown).

Participants maintained detailed food diaries for 3 days before each sample collection point, allowing for dietary intake assessment. Across the 15 participants, dietary records revealed the consumption of 62 distinct plants, with eight genera encompassing more than one type of consumed item. For example, the *Citrus* genus, within the family *Rutaceae*, included items such as oranges, clementines, grapefruits, and pomelos. Overall, participants reported consuming plants from 41 different genera. Sequencing of the *trn*L gene identified 55 plant genera (Figure 1). This discrepancy is primarily attributed to the detection of herbs such as oregano, basil, and thyme, which were not recorded in the dietary records. As expected, the *trn*L method was unable to detect certain items like coffee, cocoa, and mushrooms.

Furthermore, given that the patients were permitted to consume fish no more than twice a week, we assessed whether any participants consumed porcine or bovine meat products during the intervention. As expected, nine patients tested positive for consumption of pig, cattle, or both at baseline. Notably, one patient tested positive for consumption of bovine products at week 4. Despite this, the patient significantly increased their plant intake throughout the intervention, and exclusion of this patient from the analysis did not result in any significant differences in bacterial diversity (data not shown). Additionally, two other patients showed vague positivity for bovine consumption. Due to the low abundant signals, we attributed this to the ingestion of bovine by-products such as gelatin (42), rather than direct consumption of bovine meat.

### 3.3 A plant-based diet reverts bacterial hallmark symptom of CD

**Figure 2:**
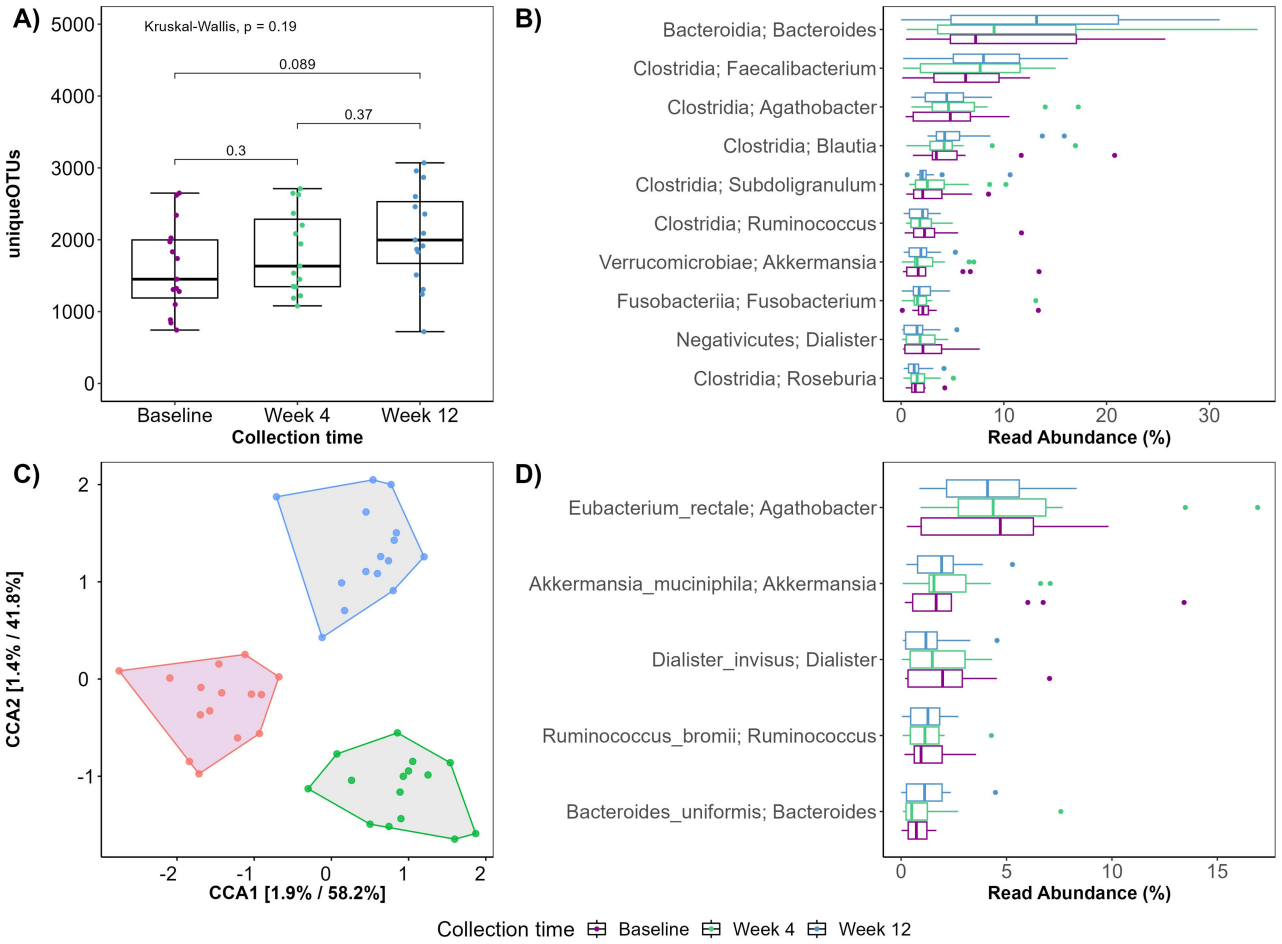
(A) Boxplot displaying observed plant genera richness. Each dot represents a sample. The boxplots show the median, the first quantile, and the third quantile. The whiskers extend to 1.5 interquartile range (IQR). *p*- values are determined using the Wilcoxon rank sum test and Kruskal-Wallis. (B,D) Boxplot displaying top 10 genera (B) and top 5 species (D) exhibiting the largest change in relative abundance according to collection time. (C) Ordination plot using canonical correspondence analysis constrained on collection time.

Sequencing of the V1V8 region of the 16S rRNA gene resulted in a total of 3,108,001 reads with an average sequencing depth of 64,750 ± 32,269 (mean ± SD) before filtering. The patients exhibited a high inter-individual difference in the microbial composition at baseline. As the intervention progressed, a reduction in this difference was observed and post-intervention, patients exhibited a more similar microbiota composition (data not shown), suggesting a convergent microbial response to the PBD. Alpha diversity plots were created to investigate the ability of a PBD to counteract the decreased microbial diversity observed in CD (43) and although not reaching the significance threshold (*p* < 0.05), the alpha diversity tended to increase during the entire intervention (baseline – week 12, *p* = 0.089; Figure 2A).

Compared to baseline, specific bacterial genera had changed abundances at week 12 (Figure 2B). Of the 10 genera exhibiting the highest relative abundance change, six belonged to the *Clostridia* class. Three genera of the *Clostridia* class; *Faecalibacterium, Blautia* and *Subdoligranulum* increased, while *Agathobacter, Roseburia* and *Rominococcus* appeared unaffected by the 12-week intervention. Most pronounced was the increase in *Faecalibacterium* and *Bacteriodes*. Notably, at species level, the known SCFA producers *Rominococcus bromii*, and *Bacteroides uniformis* increased during the intervention. *Akkermansia muciniphila* also increased while *Eubacterium rectale* and *Dialister invisus* decreased (Figure 2D).

Generally, an overall shift in diversity and microbial composition was observed during the 12-week intervention (Figure 2C). Constrained ordination revealed that 1.9 and 1.4% of the variation in PC1 and PC2 respectively, could be explained by the time the fecal sample was collected (baseline, week 4 or week 12).

### 3.4 Entry diet influences the effect of PBD on alpha diversity

**Figure 3:**
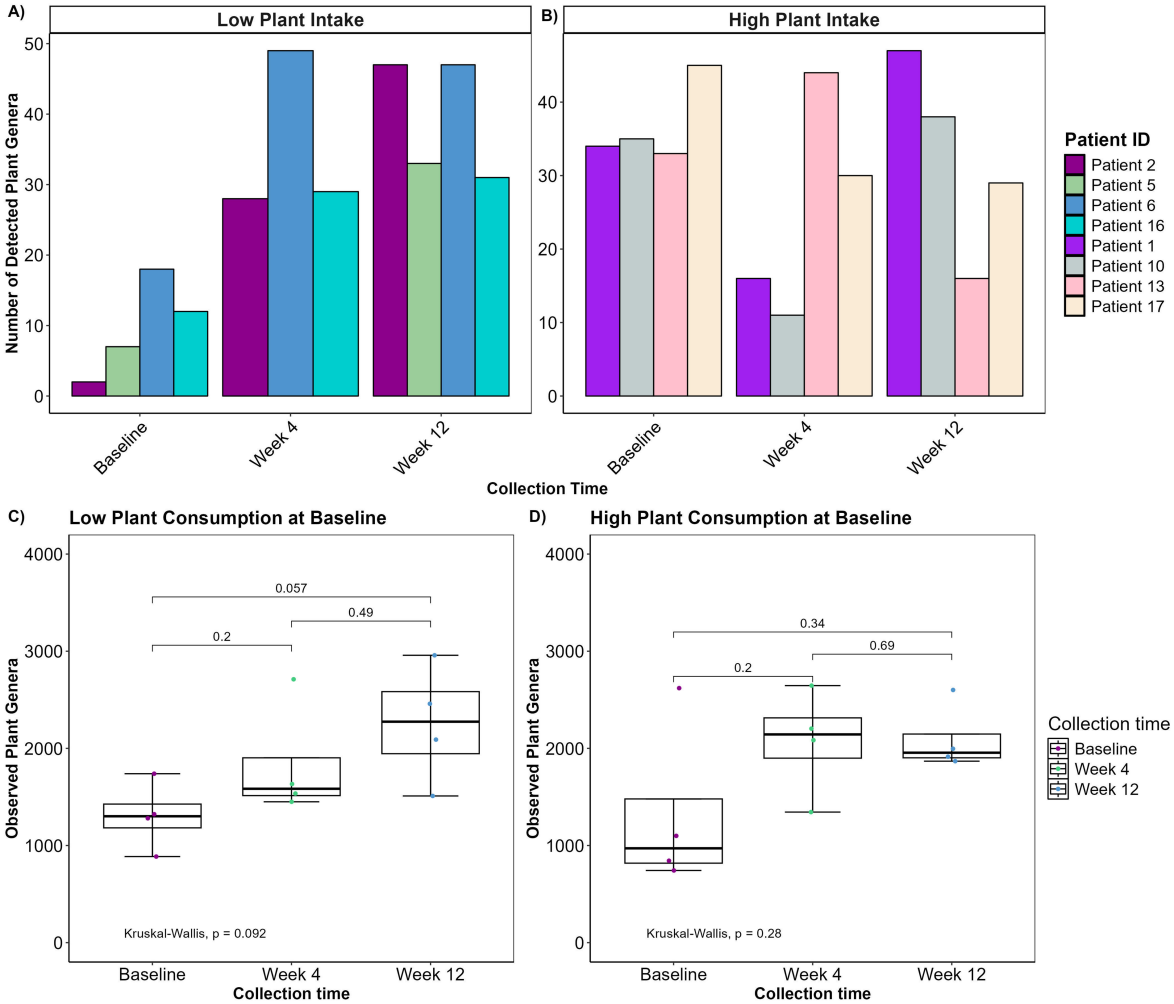
(A,B) Bar plots depicting number of detected plant genera over the course of the intervention in patients with high initial plant intake (A, *n* = 4) and low initial plant intake (B, *n* = 4). (C,D) Boxplot displaying observed plant genera richness for low (C) and high (D) initial plant intake. The boxplots show the median, the first quantile, and the third quantile. The whiskers extend to 1.5 interquartile range (IQR). *p*- values are determined using the Wilcoxon rank sum test and Kruskal-Wallis. No plants were detected in one patient with low plant intake at week 4.

Given that the patients entered the study with markedly different dietary habits, we considered whether varying initial consumption of plant genera might influence their microbial diversity in response to a PBD. Therefore, based on their initial plant genera intake, patients consuming fewer than 20 plant genera (*N* = 4) and patients consuming more than 32 plant genera (*N* = 4) were categorized into two subgroups. Patients with a low plant genera intake at baseline exhibited a significant increase in their plant genera consumption during the intervention, whereas those with a high plant intake at baseline showed only a modest change in their overall plant genera intake with some consuming fewer plant genera at week 4 or 12 (Figures 3A,B). It was then tested how this difference affected microbial diversity in the same patients (Figures 3C,D). Notably, patients with initially low plant intake exhibited a greater overall increase in alpha diversity (*p* = 0.092) compared to those with high baseline plant intake (*p* = 0.28). This suggests that the initial dietary pattern may influence the effectiveness of dietary interventions on microbial diversity.

### 3.5 PBD decreases fecal calprotectin levels and is related to microbial composition

**Figure 4:**
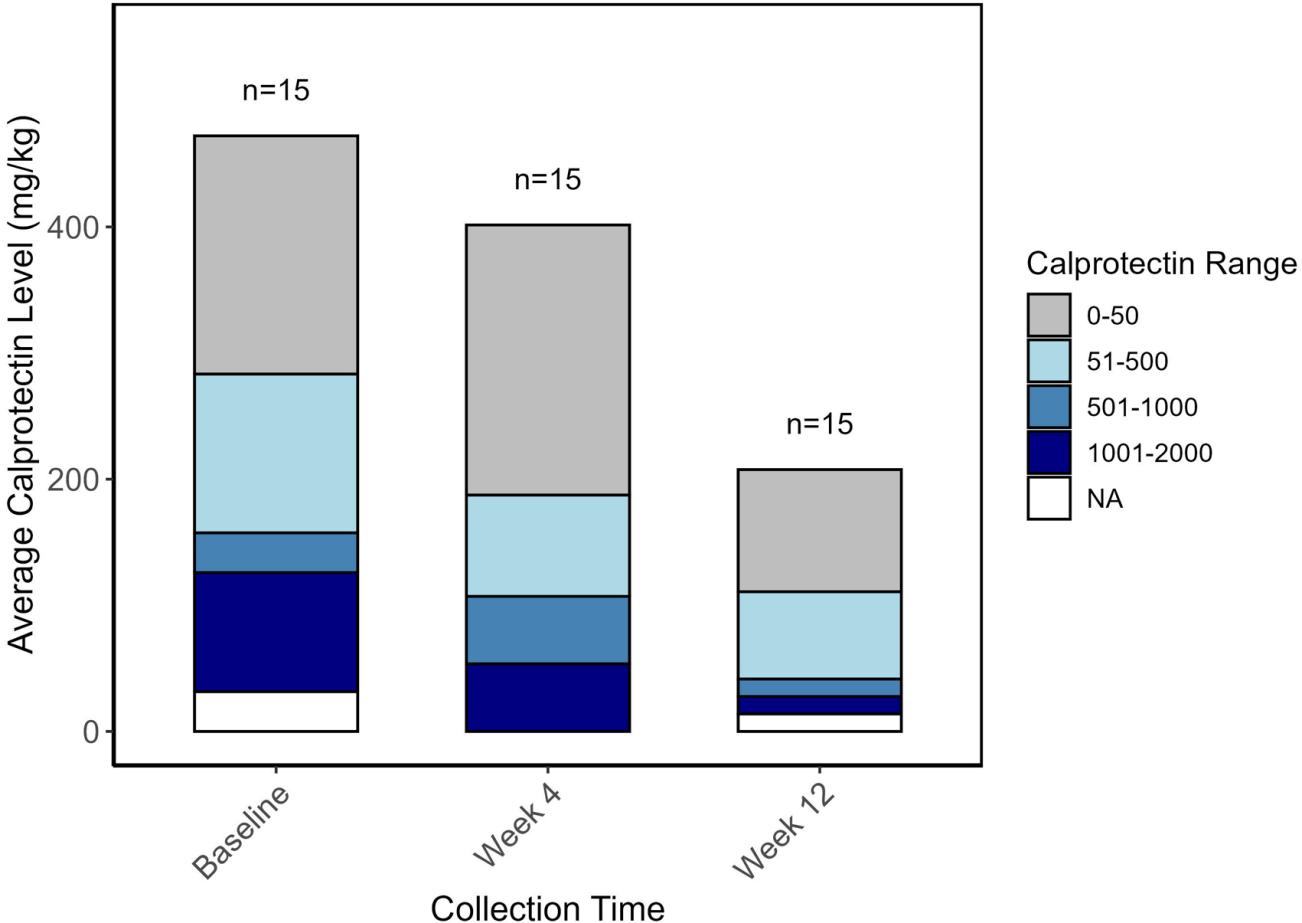
Bar plot representing the average fecal calprotectin levels (mg/kg) at three time points: Baseline, Week 4, and Week 12. The height of the bars represents the overall average calprotectin levels at each time point. The stacked bars indicate the distribution of patients across different calprotectin level ranges: 0–50 mg/kg (gray), 51–500 mg/kg (light blue), 501–1,000 mg/kg (steel blue), and 1,001–2000 mg/kg (navy blue). NA (white) represents missing values. The numbers above each bar indicate the sample size (*n* = 15) at each time point.

Fecal calprotectin level was obtained for each patient at baseline, week 4, and week 12 of the intervention. Notably, the average calprotectin level decreased from 472 mg/kg at baseline to 207 mg/kg at week 12 (Figure 4). When determining linear associations between the microbial community and calprotectin levels across the three collection points, three bacterial species appeared to be associated with measured calprotectin levels. *Ruminococcus faecis* and a bacterium belonging to the *Lachnospiraceae* family were positively correlated with calprotectin levels (False Discovery Rate (FDR): 1.283e-1 and FDR: 1.018e-1) whereas a bacterium belonging to the UCG.005 genus, within the *Oscillospiraceae* family, was negatively correlated with calprotectin levels (FDR: 1.283e-1) (data not shown).

## 4 Discussion

This study aimed to advance our understanding of the impact of PBD on CD by combining traditional dietary assessments and advanced molecular techniques. Despite the limitations of a relatively small dataset, the findings offer significant insights into several key areas regarding the enteric microbiome and its relationship with diet.

One of the study’s key contributions is the demonstration of a reliable method for measuring plant residue diversity in fecal samples as an objective metric for assessing adherence to a PBD. The use of *trn*L amplicon sequencing overcomes to some extent the limitations of self-reported dietary data, which is often prone to misreporting (41). This is evident from the discrepancy observed between the 41 plant genera reported in dietary records and the 55 identified through *trn*L sequencing, providing a more accurate reflection of food items ingested. The ability to monitor changes in plant intake and detect porcine and bovine consumption validates the effectiveness of the dietary intervention, ensuring that the observed changes in the microbiota and clinical parameters are attributable to the PBD rather than confounding dietary factors (e.g., low diversity diet, meat consumption etc.). More importantly, the absence of a significant effect might also be explained by inconsistent adherence to the diet. This underlines the importance of precise dietary monitoring, as incomplete adherence could mask potential benefits of the PBD and lead to an underestimation of its true impact on the microbiota and clinical outcomes.

The rapid acceptance and adherence to the PBD led to a marked increase in both *trn*L-detected plant diversity and plant genera recorded in dietary records, with a concurrent notable increase in bacterial diversity. This is significant, as reduced gut microbial diversity is a hallmark of inflammatory bowel disease (IBD) pathogenesis (4, 44). While the observed increase in alpha diversity during the intervention did not reach statistical significance, it suggests a potential trend toward the restoration of microbial diversity in CD patients. This finding aligns with emerging evidence that dietary modifications emphasizing plant-based foods can have beneficial effects on CD symptoms and disease progression (4, 27, 29).

The identification of specific bacterial genera and species exhibiting changes in abundance following the PBD provides insights into potential mechanisms underlying its therapeutic effects in CD. An increase in beneficial genera such as *Faecalibacterium* and *Bacteroides*, both of which have been reported to be underrepresented in patients with CD (45–47), along with known SCFA producers like *Ruminococcus bromii* and *Bacteroides uniformis*, suggests a shift toward a gut microbiota profile associated with improved intestinal health and immune regulation (27, 44). However, an unexpected decrease in *Eubacterium rectale* and *Dialister invisus*, both known SCFA producers with roles in mitigating TNF-related inflammation (48), was observed. Given that these species are typically expected to increase with elevated fiber intake (49), this finding requires further investigation to understand the specific factors influencing their abundance in the context of a PBD.

The intervention resulted in a reduction in fecal calprotectin levels, a commonly used non-invasive biomarker for monitoring IBD (50), from an average of 472 mg/kg at baseline to 207 mg/kg at the end of the intervention. Although relatively high FDRs limit the strength of the linear associations identified between specific bacterial species and calprotectin levels, the results support the hypothesis that dietary modifications can influence gut microbiota composition and, consequently, modulate inflammatory processes in CD patients. The bacterial species associated with calprotectin levels are particularly intriguing as potential biomarkers for inflammation in CD.

While *trn*L sequencing is a powerful tool, it is not without its limitations (41). Certain plant items, such as coffee, cocoa, and mushrooms do not contain chloroplast and are therefore not detectable. Furthermore, there are no current guidelines on the duration specific plants can be detected in fecal samples after ingestion. This study used dietary records for 3 days before sampling. In future studies, it might be relevant to collect fecal samples alongside dietary recording to more accurately benchmark *trn*L amplicon sequencing. Structured databases for comprehensive dietary assessment with species-level identification are also needed. Lastly, regarding *trn*L sequencing, it is important to emphasize that while *trn*L is valuable for identifying plant intake, it remains qualitative in nature and does not provide a quantitative measure of how much of each plant is consumed (41). This limitation can be crucial in dietary studies, as both the diversity and the amount of plant intake can be equally important in evaluating the impact of diet on health outcomes (51). Furthermore, while the trend toward increased microbial diversity is promising, the lack of statistical significance is likely due to the small sample size and high inter-individual variability, which limits the power to detect significant effects. Based on a power calculation with similar study setup and the observed alpha diversity changes (600, SD: 900) found in this pilot study it was estimated that 35 participants would be needed to achieve 80% power with a significance level of 0.05 (52). To account for dropouts, we recommend recruiting 40 participants in future studies. However, while some findings did not reach statistical significance, they reveal important trends on the dietary effects and required lengths of the dietary intervention to assess effects of the plant-based diet on gut microbiota and inflammation.

## 5 Conclusion

In summary, this study not only establishes a method for directly identifying ingested plant items and adherence to a PBD but also identifies critical clinical and microbiological parameters for evaluating dietary interventions. The findings contribute to a more nuanced understanding of how dietary changes impact CD and highlight the need for further research to refine these methods and explore their broader applications in clinical practice. Additionally, the study provides preliminary evidence that a PBD can positively influence the gut microbiome and reduce inflammation in CD patients. These results highlight the potential of dietary interventions as adjunctive therapies in the management of CD.

## Data Availability

The datasets presented in this study can be found in online repositories. The names of the repository/repositories and accession number(s) can be found at: https://www.ebi.ac.uk/ena, PRJEB80522; https://www.ebi.ac.uk/ena, PRJEB79272.

## Footnotes

^1^ https://github.com/nanoporetech/dorado

## References

[1] Baumgart DC Sandborn WJ. Crohn’s disease. Lancet. (2012) 380:1590–605. doi: 10.1016/S0140-6736(12)60026-9 22914295

[2] Schirmer M Garner A Vlamakis H Xavier RJ. Microbial genes and pathways in inflammatory bowel disease. Nat Rev Microbiol. (2019) 17:497–511. doi: 10.1038/s41579-019-0213-6, PMID: 31249397 PMC6759048

[3] Petersen C Round JL. Microreview defining dysbiosis and its influence on host immunity and disease. Cell Microbiol. (2014) 16:1024–33. doi: 10.1111/cmi.12308, PMID: 24798552 PMC4143175

[4] Venegas DP De la Fuente MK Landskron G González MJ Quera R Dijkstra G. Short chain fatty acids (SCFAs)-mediated gut epithelial and immune regulation and its relevance for inflammatory bowel diseases. Front Immunol. (2019) 10:277. doi: 10.3389/FIMMU.2019.00277, PMID: 30915065 PMC6421268

[5] Cai Z Wang S Li J. Treatment of inflammatory bowel disease: a comprehensive review. Front Med (Lausanne). (2021) 8:765474. doi: 10.3389/fmed.2021.765474, PMID: 34988090 PMC8720971

[6] Antoniussen CS Rasmussen HH Holst M Lauridsen C. Reducing disease activity of inflammatory bowel disease by consumption of plant-based foods and nutrients. Front Nutr. (2021) 8:733433. doi: 10.3389/fnut.2021.733433, PMID: 34957174 PMC8696360

[7] Lane ER Zisman TL Suskind DL. The microbiota in inflammatory bowel disease: current and therapeutic insights. J Inflamm Res. (2017) 10:63–73. doi: 10.2147/JIR.S116088, PMID: 28652796 PMC5473501

[8] Bolte LA Vich Vila A Imhann F Collij V Gacesa R Peters V. Gut microbiota long-term dietary patterns are associated with pro-inflammatory and anti-inflammatory features of the gut microbiome. Gut. (2021) 70:1287–98. doi: 10.1136/gutjnl-2020-322670, PMID: 33811041 PMC8223641

[9] Kim MH Park JH Yanagisawa M Kim CH Kim CH. Short-chain fatty acids activate GPR41 and GPR43 on intestinal epithelial cells to promote inflammatory responses in mice. Gastroenterology. (2013) 145:56. doi: 10.1053/j.gastro.2013.04.056, PMID: 23665276

[10] Joossens M Huys G Cnockaert M de Preter V Verbeke K Rutgeerts P. Dysbiosis of the faecal microbiota in patients with Crohn’s disease and their unaffected relatives. Gut. (2011) 60:631–7. doi: 10.1136/GUT.2010.223263, PMID: 21209126

[11] Huda-Faujan N Abdulamir AS Fatimah AB Anas OM Shuhaimi M Yazid AM. The impact of the level of the intestinal short chain fatty acids in inflammatory bowel disease patients versus healthy subjects. Open Biochem J. (2010) 4:53–8. doi: 10.2174/1874091X01004010053, PMID: 20563285 PMC2887640

[12] Christ A Lauterbach M Latz E. Western diet and the immune system: an inflammatory connection. Immunity. (2019) 51:794–811. doi: 10.1016/J.IMMUNI.2019.09.020, PMID: 31747581

[13] Temba GS Kullaya V Pecht T Mmbaga BT Aschenbrenner AC Ulas T. Urban living in healthy Tanzanians is associated with an inflammatory status driven by dietary and metabolic changes. Nat Immun. (2021) 22:287–300. doi: 10.1038/s41590-021-00867-8, PMID: 33574617

[14] Wu D Wang X Yang X Gu L McGeachy M Liu X. Temporary consumption of western diet trains the immune system to reduce future gut inflammation. iScience. (2023) 26:106915. doi: 10.1016/J.ISCI.2023.106915, PMID: 37305694 PMC10250831

[15] Ananthakrishnan AN Khalili H Konijeti GG Higuchi LM de Silva P Korzenik JR. A prospective study of long-term intake of dietary fiber and risk of Crohn’s disease and ulcerative colitis. Gastroenterology. (2013) 145:970–7. doi: 10.1053/J.GASTRO.2013.07.050, PMID: 23912083 PMC3805714

[16] Chiba M Nakane K Komatsu M. Westernized diet is the Most ubiquitous environmental factor in inflammatory bowel disease. Perm J. (2019) 23:18–107. doi: 10.7812/TPP/18-107, PMID: 30624192 PMC6326567

[17] Beam A Clinger E Hao L. Effect of diet and dietary components on the composition of the gut microbiota. Nutrients. (2021) 13:2795. doi: 10.3390/nu13082795 34444955 PMC8398149

[18] Godala M Gaszyńska E Zatorski H Małecka-Wojciesko E. Godala dietary interventions in inflammatory bowel disease. Nutrients. (2022) 14:4261. doi: 10.3390/nu14204261 36296945 PMC9607252

[19] Reddavide R Rotolo O Caruso MG Stasi E Notarnicola M Miraglia C. The role of diet in the prevention and treatment of inflammatory bowel diseases. Acta Biomed. (1885) 89:60–75. doi: 10.23750/abm.v89i9-S.7952, PMID: 30561397 PMC6502201

[20] Berni Canani R Terrin G Borrelli O Romano MT Manguso F Coruzzo A. Short-and long-term therapeutic efficacy of nutritional therapy and corticosteroids in paediatric Crohn’s disease. Dig Liver Dis. (2006) 38:381–7. doi: 10.1016/j.dld.2005.10.005 16301010

[21] Borrelli O Cordischi L Cirulli M Paganelli M Labalestra V Uccini S. Polymeric diet alone versus corticosteroids in the treatment of active pediatric Crohn’s disease: a randomized controlled open-label trial. Clin Gastroenterol Hepatol. (2006) 4:744–53. doi: 10.1016/j.cgh.2006.03.010, PMID: 16682258

[22] Grover Z Muir R Reilly C Lewindon PJ Lewindon PJ. Early mucosal healing with exclusive enteral nutrition is associated with improved outcomes in newly diagnosed children with luminal Crohn’s disease. J Crohns Colitis. (2016) 10:1159–64. doi: 10.1093/ECCO-JCC/JJW075, PMID: 26980840

[23] Levine A Wine E Assa A Sigall Boneh R Shaoul R Kori M. Crohn’s disease exclusion diet plus partial enteral nutrition induces sustained remission in a randomized controlled trial. Gastroenterology. (2019) 157:440–450.e8. doi: 10.1053/J.GASTRO.2019.04.021, PMID: 31170412

[24] Miller T Suskind DL. Exclusive enteral nutrition in pediatric inflammatory bowel disease. Curr Opin Pediatr. (2018) 30:671–6. doi: 10.1097/MOP.0000000000000660, PMID: 30004946

[25] Sigall Boneh R van Limbergen J Wine E Assa A Shaoul R Milman P. Dietary therapies induce rapid response and remission in pediatric patients with active Crohn’s disease. Clin Gastroenterol Hepatol. (2021) 19:752–9. doi: 10.1016/J.CGH.2020.04.006, PMID: 32302709

[26] Suskind DL Lee D Kim YM Wahbeh G Singh N Braly K. The specific carbohydrate diet and diet modification as induction therapy for pediatric Crohn’s disease: a randomized diet controlled trial. Nutrients. (2020) 12:1–23. doi: 10.3390/NU12123749, PMID: 33291229 PMC7762109

[27] Olendzki B Bucci V Cawley C Maserati R McManus M Olednzki E. Dietary manipulation of the gut microbiome in inflammatory bowel disease patients: pilot study. Gut Microbes. (2022) 14:244. doi: 10.1080/19490976.2022.2046244, PMID: 35311458 PMC8942410

[28] Chiba M Ishii H Komatsu M. Recommendation of plant-based diets for inflammatory bowel disease. Transl Pediatr. (2019) 8:23–7. doi: 10.21037/TP.2018.12.02, PMID: 30881895 PMC6382506

[29] Chiba M Morita N. Incorporation of plant-based diet surpasses current standards in therapeutic outcomes in inflammatory bowel disease. Metabolites. (2023) 13:332. doi: 10.3390/METABO13030332, PMID: 36984772 PMC10051661

[30] Arvidsson LB Lauridsen C Mikkelsen S Rasmussen HH Cetin Z Østergaard SK. A plant-based diet is feasible in patients with Crohn’s disease. Clin Nutr. (2024) 64:28–36. doi: 10.1016/J.CLNESP.2024.09.003, PMID: 39251088

[31] Ravelli MN Schoeller DA. Traditional self-reported dietary instruments are prone to inaccuracies and new approaches are needed. Front Nutr. (2020) 7:538983. doi: 10.3389/FNUT.2020.00090/BIBTEX PMC7350526 32719809

[32] Best WR Becktel JM Singleton JW Kern F. Development of a Crohn’s disease activity index: National Cooperative Crohn’s disease study. Gastroenterology. (1976) 70:439–44. doi: 10.1016/S0016-5085(76)80163-1 1248701

[33] Schacksen PS Østergaard SK Eskildsen MH Nielsen JL. Complete pipeline for Oxford Nanopore technology amplicon sequencing (ONT-AmpSeq): From pre-processing to creating an operational taxonomic unit table. FEBS Open Bio. (2024) 1:13868. doi: 10.1002/2211-5463.13868 PMC11532972 39109544

[34] Quast C Pruesse E Yilmaz P Gerken J Schweer T Yarza P. The SILVA ribosomal RNA gene database project: improved data processing and web-based tools. Nucleic Acids Res. (2013) 41:D590–6. doi: 10.1093/nar/gks1219, PMID: 23193283 PMC3531112

[35] Altschul SF Gish W. [27] local alignment statistics In: Doolittle RF, editor. Computer Methods for Macromolecular Sequence Analysis, vol. 266. Amsterdam, Netherlands: Elsevier Science (1996). 460–80. 10.1016/s0076-6879(96)66029-7 8743700

[36] R Core Team. R: The R project for statistical computing. Vienna: R Core Team (2020).

[37] RStudio Team. RStudio: integrated development for R. Boston: RStudio, PBC (2020).

[38] Andersen KS Kirkegaard RH Karst SM Albertsen M. ampvis2: an R package to analyse and visualise 16S rRNA amplicon data. bioRxiv. (2018) 2018:299537. doi: 10.1101/299537

[39] Wickham H. ggplot2: Elegent graphics for data analysis. New York: Springer Verlag (2016).

[40] Mallick H Rahnavard A McIver LJ Ma S Zhang Y Nguyen LH. Multivariable association discovery in population-scale meta-omics studies. PLoS Comput Biol. (2021) 17:e1009442. doi: 10.1371/JOURNAL.PCBI.1009442 34784344 PMC8714082

[41] Taberlet P Coissac E Pompanon F Gielly L Miquel C Valentini A. Power and limitations of the chloroplast trnL (UAA) intron for plant DNA barcoding. Nucleic Acids Res. (2007) 35:e14. doi: 10.1093/NAR/GKL938, PMID: 17169982 PMC1807943

[42] Mariod AA Fadul H. Review: gelatin, source, extraction and industrial applications. Acta Sci Pol Technol Aliment. (2013) 12:135–47.

[43] Manichanh C Rigottier-Gois L Bonnaud E Gloux K Pelletier E Frangeul L. Reduced diversity of faecal microbiota in Crohn’s disease revealed by a metagenomic approach. Gut. (2006) 55:205–11. doi: 10.1136/GUT.2005.073817, PMID: 16188921 PMC1856500

[44] Khan I Ullah N Zha L Bai Y Khan A Zhao T. Alteration of gut microbiota in inflammatory bowel disease (IBD): cause or consequence? IBD treatment targeting the gut microbiome. Pathogens. (2019) 8:126. doi: 10.3390/PATHOGENS8030126 31412603 PMC6789542

[45] Björkqvist O Seifert M Brislawn C Jansson J Engstrand L Rangel I. Alterations in the relative abundance of *Faecalibacterium prausnitzii* correlate with changes in fecal calprotectin in patients with ileal Crohn’s disease: a longitudinal study. Scand J Gastroenterol. (2019) 54:577–85. doi: 10.1080/00365521.2019.1599417, PMID: 31104514

[46] Morgan XC Tickle TL Sokol H Gevers D Devaney KL Ward DV. Dysfunction of the intestinal microbiome in inflammatory bowel disease and treatment. Genome Biol. (2012) 13:R79. doi: 10.1186/gb-2012-13-9-r79, PMID: 23013615 PMC3506950

[47] Moustafa A Li W Anderson EL Wong EHM Dulai PS Sandborn WJ. Genetic risk, dysbiosis, and treatment stratification using host genome and gut microbiome in inflammatory bowel disease. Clin Transl Gastroenterol. (2018) 9:e132. doi: 10.1038/ctg.2017.58, PMID: 29345635 PMC5795019

[48] Lu H Xu X Fu D Gu Y Fan R Yi H. Butyrate-producing *Eubacterium rectale* suppresses lymphomagenesis by alleviating the TNF-induced TLR4/MyD88/NF-κB axis. Cell Host Microbe. (2022) 30:1139–1150.e7. doi: 10.1016/J.CHOM.2022.07.003, PMID: 35952646

[49] Vinelli V Biscotti P Martini D del Bo’ C Marino M Meroño T. Effects of dietary fibers on short-chain fatty acids and gut microbiota composition in healthy adults: a systematic review. Nutrients. (2022) 14:2559. doi: 10.3390/nu14132559, PMID: 35807739 PMC9268559

[50] Walsham NE Sherwood RA. Fecal calprotectin in inflammatory bowel disease. Clin Exp Gastroenterol. (2016) 9:21–9. doi: 10.2147/CEG.S51902, PMID: 26869808 PMC4734737

[51] Sun C Zhang WS Jiang CQ Jin YL Zhu T Zhu F. Quantity and variety in fruit and vegetable consumption and mortality in older Chinese: a 15-year follow-up of a prospective cohort study. J Nutr. (2023) 153:2061–72. doi: 10.1016/J.TJNUT.2023.03.021, PMID: 36963500

[52] Gupta KK Attri JP Singh A Kaur H Kaur G. Basic concepts for sample size calculation: critical step for any clinical trials! Saudi J Anaesth. (2016) 10:328–31. doi: 10.4103/1658-354X.174918, PMID: 27375390 PMC4916819

[53] Lane D. J. Pace B Olsen GJ Stahl DA Sogin ML Pace NR. (1985) . Rapid determination of 16S ribosomal RNA sequences for phylogenetic analyses (reverse transcriptase/dideoxynudeotide). 82:6955–6959. doi: 10.1073/pnas.82.20.6955, PMID: 2413450 PMC391288

[54] Weisburg WG Barns SM Pelletier DA Lane DJ. 16S ribosomal DNA amplification for phylogenetic study. J Bacteriol. (1991) 173:697–703. doi: 10.1128/JB.173.2.697-703.1991, PMID: 1987160 PMC207061

[55] Matsunaga T Chikuni K Tanabe R Muroya S Shibata K Yamada J. A quick and simple method for the identification of meat species and meat products by PCR assay. Meat Sci. (1999) 51:143–8. doi: 10.1016/S0309-1740(98)00112-0, PMID: 22061698

